# Novel loss-of-function variants in *TRIO* are associated with neurodevelopmental disorder: case report

**DOI:** 10.1186/s12881-020-01159-y

**Published:** 2020-11-10

**Authors:** Laura Schultz-Rogers, Karthik Muthusamy, Filippo Pinto e Vairo, Eric W. Klee, Brendan Lanpher

**Affiliations:** 1 Center for Individualized Medicine, Mayo Clinic, Rochester, MN USA; 2 Department of Clinical Genomics, Mayo Clinic, Rochester, MN USA

**Keywords:** *TRIO* gene, Autism, Macrocephaly, Microcephaly, Cutis aplasia

## Abstract

**Background:**

Damaging variants in *TRIO* have been associated with moderate to severe neurodevelopmental disorders in humans. While recent work has delineated the positional effect of missense variation on the resulting phenotype, the clinical spectrum associated with loss-of-function variation has yet to be fully defined.

**Case presentation:**

We report on two probands with novel loss-of-function variants in *TRIO*. Patient 1 presents with a severe neurodevelopmental disorder and macrocephaly. The *TRIO* variant is inherited from his affected mother. Patient 2 presents with moderate developmental delays, microcephaly, and cutis aplasia with a frameshift variant of unknown inheritance.

**Conclusions:**

We describe two patients with neurodevelopmental disorder, macro/microcephaly, and cutis aplasia in one patient. Both patients have loss-of-function variants, helping to further characterize how these types of variants affect the phenotypic spectrum associated with *TRIO*. We also present the third reported case of autosomal dominant inheritance of a damaging variant in *TRIO*.

## Background

The human gene *TRIO* (triple functional domain) encodes a guanine nucleotide exchange factor (GEF) that facilitates the activation of Rho GTPases such as RAC1 [1–3]. Rho GTPases in turn regulate actin cytoskeleton dynamics and thus play an important role in many neurodevelopmental processes such as migration of neural progenitors and neurons during neurogenesis, synapse formation, and axon outgrowth [4–7]. The TRIO protein contains several catalytic domains including two GEF domains. The GEF1 domain activates RAC1 and RHOG while the GEF2 domain activates RHOA [8–11]. The protein also contains a c-terminal serine/threonine kinase domain [3]. *TRIO* is a large gene with 57 exons and multiple tissue-specific isoforms, including several different isoforms expressed in the mammalian brain [12–15]. Studies have shown that expression of *TRIO* peaks in the brain in the late prenatal period [16] supporting a role for this gene in neurodevelopmental processes. The importance of this gene in mammalian neurodevelopment has been further highlighted by studies in mice which have shown complete knockout of the gene to be embryonic lethal, while nervous system-specific knockout mice show abnormal brain morphology, reduced brain size, and altered learning ability [17–21]. Additionally, it has been shown in vitro that *TRIO* is important in regulating synaptic function through regulation of AMPAr endocytosis at CA1 excitatory synapses [22].

Several patients have been identified with rare or novel variants in *TRIO* from large cohort studies examining whole exome sequencing data from patients with generalized phenotypes such as intellectual disability (ID), neurodevelopmental disorders, schizophrenia, childhood speech apraxia, autism spectrum disorder (ASD), microcephaly, and epilepsy with intellectual disability [16, 22–35]. Recently, however, several *TRIO*-specific cohorts with functional studies have been described that have helped elucidate the role of pathogenic variation in this gene in human disease [16, 22, 36–38]. Consistently reported phenotypes described in association with variants in this gene include global developmental delay, speech disorder, ID and learning disabilities, and macro- or microcephaly. Additional phenotypes include behavioral issues, dysmorphic facies, skeletal/hand anomalies, feeding difficulties, insensitivity to pain, seizures, tremor, ataxia, abnormal brain MRI, hypotonia, dental anomalies, congenital heart defects, urinary incontinence, and ocular defects.

**Fig. 1:**
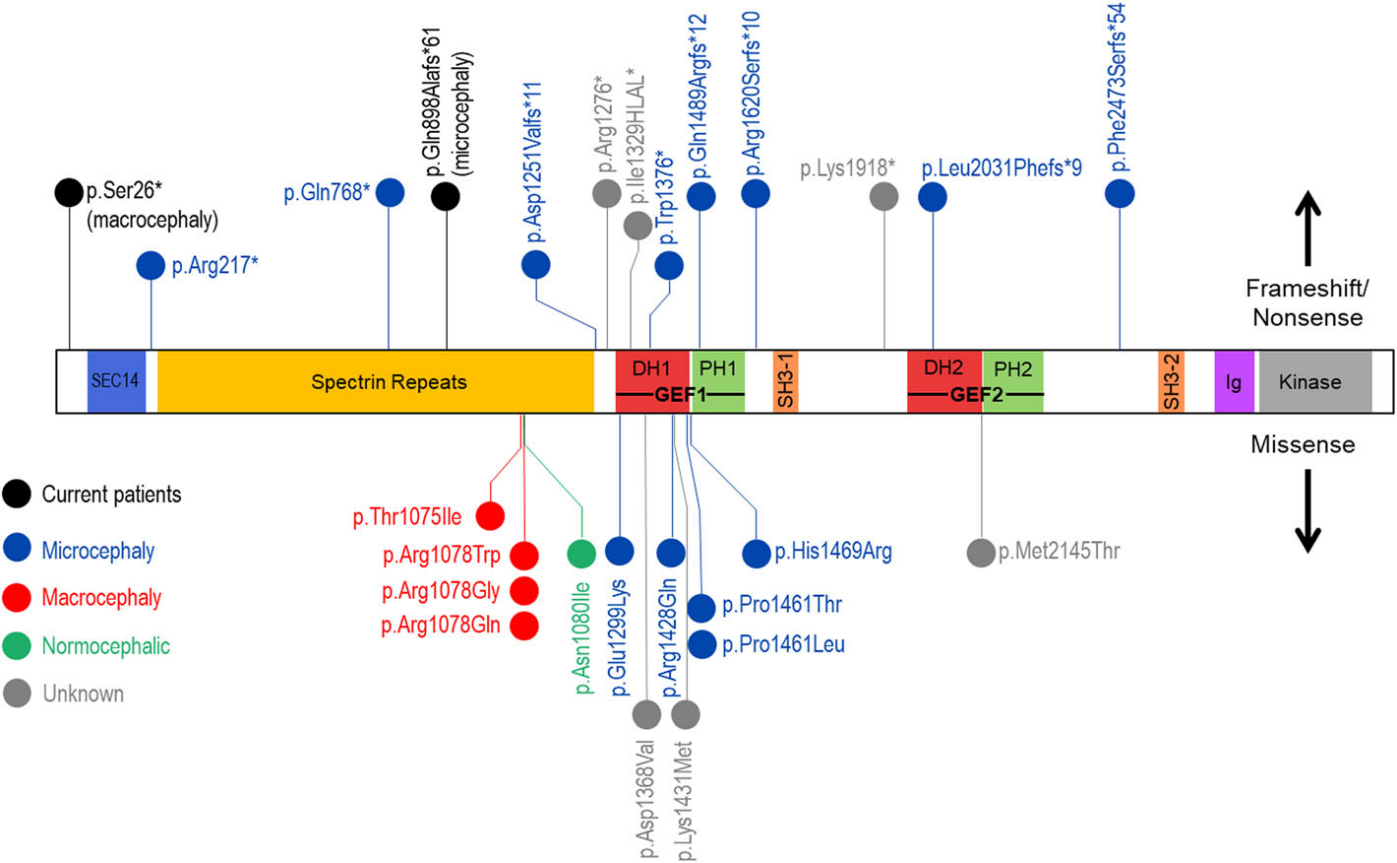
*Variants associated with neurodevelopment disorder in TRIO*. Schematic of protein domains of human TRIO (NM_NM_007118.3). Variants discussed in this report are in black. Variants associated with microcephaly are in blue; microcephaly-associated frameshift/nonsense variants span the gene, while microcephaly-associated missense variants cluster in the GEF1 domain. Macrocephaly-associated missense variants cluster in the Spectrin repeats domain. With the exception of current patient 1 described here, all frameshift/nonsense variants have to date been associated with microcephaly

Interestingly, it has become clear that there is a strong phenotype/genotype correlation specific to missense variants in this gene. Several missense variants that cluster in the spectrin repeat domain have been functionally shown to lead to increased RAC1 activation and enhanced neurite outgrowth and lamellipodia formation [36] (Fig. 1). Strikingly, all of the patients with missense variants in this region demonstrated moderate to severe ID and macrocephaly. Of note, Pengelly et al. also reported a patient with a p.Asn1080Ile missense variant who displayed severe ID but normal head circumference [37]. Their assessment of this variant in vitro did not identify an effect on the activation of RAC1, though this discrepancy with previous reports may be due to experimental design. Overall, it is hypothesized that missense variants in this domain cause hyperactivation of RAC1 leading to the observed macrocephaly phenotype. This may be due to variants affecting protein folding and thus interfering with the intermolecular inhibition of the GEF1 domain by the spectrin repeats [36, 39]. Conversely, missense variants in the RAC1 activating GEF1 domain have been shown functionally to lead to reduced overall RAC1 binding and activation, impaired formation of neurites and lamellipodia, and a reduction in AMPAR-eEPSC amplitude [16, 36–38] (Fig. 1). The phenotypes associated with these variants include mild/moderate ID and microcephaly. In contrast to variants in the spectrin domains, it is hypothesized that variants in the GEF1 domain lead to loss of normal RAC1 activation and thus the observed microcephaly. The reason for the severity of ID appears to be greater in association with spectrin variants is unclear. There has also been one report of a hypermorphic variant in the GEF2 domain in which functional studies showed increased RhoA activity [16] (Fig. 1). It is hypothesized that this domain is auto-inhibited by the PH2 domain, and variants that disrupt this inhibition lead to hyperactivity. The phenotype associated with this variant is not well defined other than schizophrenia.

While clear associations have been shown between the location of missense variants and the resulting disease phenotype, the characterization of framsehift and nonsense variants is less well established. The majority of reported patients with loss-of-function (LOF) variants with occipitofrontal circumference (OFC) data available do display microcephaly. However, the severity of ID and other associated phenotypes does not appear to be consistent. In vitro modeling of truncating variants in the GEF1 domain has shown reduced RAC1 binding and activation [36–38], while modeling of truncating variants in the downstream GEF2 domain show show no effect on RAC1 activation. An open question remains as to whether or not these frameshift/nonsense variants are actually producing a truncated protein in vivo or if the transcripts are undergoing nonsense-mediated decay (NMD) resulting in haploinsufficiency. The presence of multiple alternate transcripts could also result in a variant-specific effect on NMD based on which transcripts are affected. Of the frameshift/nonsense described in *TRIO* cohorts so far, all would be predicted to undergo NMD based on an in silico prediction tool [40]. However none of the studies mentioned above have actually tested patient samples for absence or presence of a truncated protein product.

Here we present two patients presenting to the Department of Clinical Genomics at the Mayo Clinic with moderate/severe developmental delay, facial dysmorphisms, macro- and microcephaly, and a novel phenotype of cutis aplasia in one patient. Both patients were found to have presumed LOF variants in *TRIO* (Fig. 1). Additionally, one of the two probands harbors a variant inherited from an affected mother, making this the third documented cases of autosomal dominant (AD) familial inheritance of disease associated with this gene. The data from these patients help expand the known phenotypes associated with pathogenic *TRIO* variants, provides further evidence of AD inheritance, and helps elucidate the disease spectrum associated specifically with presumed LOF variants.

## Case presentation

### Clinical description

**Fig. 2:**
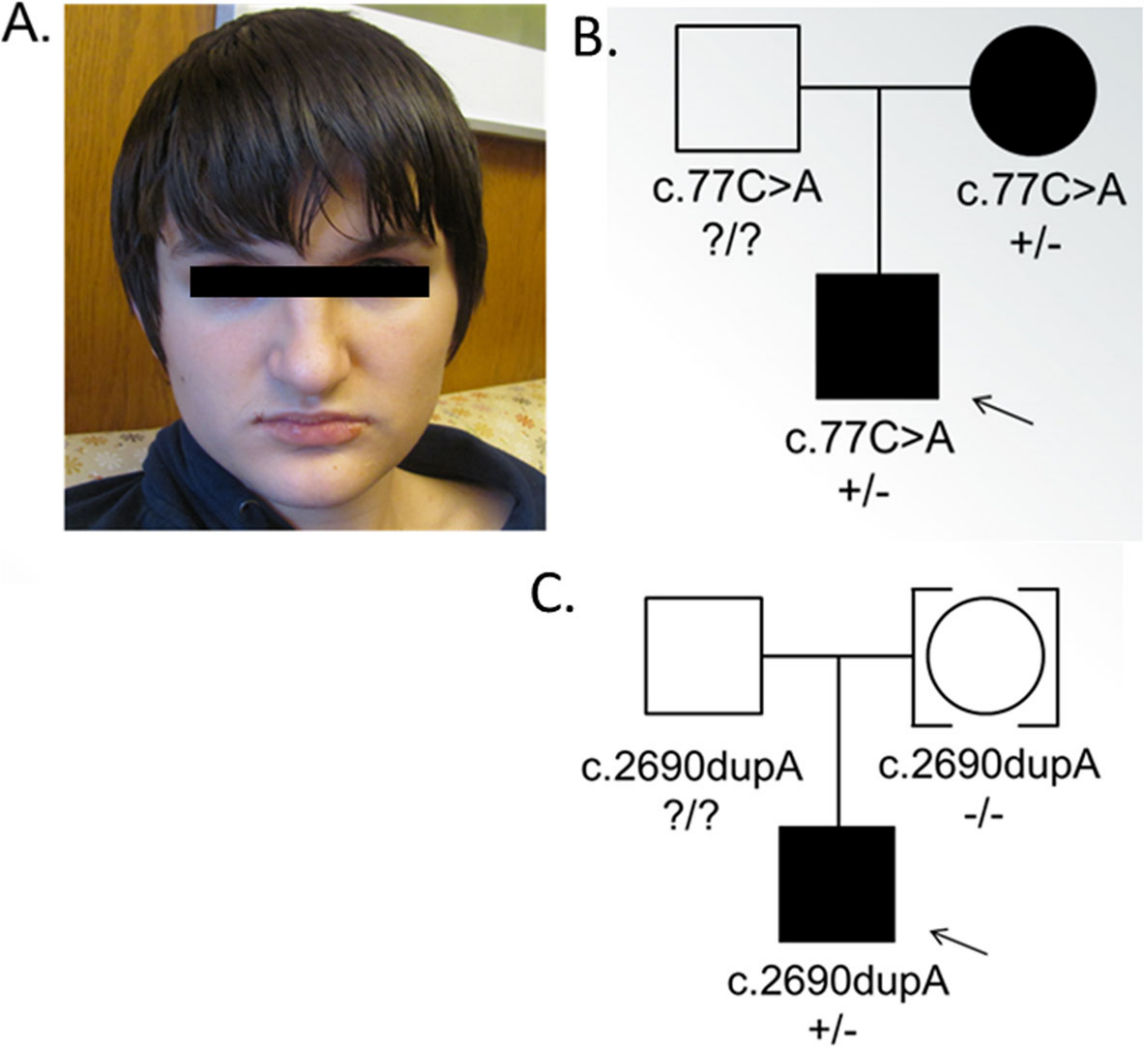
*Characteristics of patients described with novel loss-of-function variants in TRIO*. **a** Photograph of patient 1 displaying macrocephaly, broad forehead, deep set eyes, broad nasal root, hanging columella and short philtrum. **b** Pedigree for patient 1. **c**. Pedigree for patient 2

Patient 1 is a 14-year-old Caucasian boy who was born to a non-consanguineous union. He presented for genetic evaluation of autism spectrum disorder (ASD). He was born at term with appropriate birth weight, after an uncomplicated pregnancy and delivery. He had isolated delay in attaining socio-adaptive and language milestones and was noted to have repetitive behaviors. He remains nonverbal and also displays motor stereotypies such as hand flapping. Significant sleep disturbances were noticed since infancy. He was diagnosed with autism spectrum disorder at four years of age. He had febrile seizures during early childhood. He is currently in middle school with Individualized Education Program and communicates to some extent with augmented communication device. Significant behavioral anger outbursts occurred when he transitioned from elementary school to middle school which responded to symptomatic medications. There were no self-injurious behaviors or tactile sensitivities. His family history was significant for his mother with developmental delay and dyslexia as well as inflammatory bowel disease, one maternal uncle with significant developmental disability attributed to traumatic brain injury suffered at age 3, a paternal uncle with schizophrenia and a maternal 2nd cousin with autism spectrum disorder (Fig. 2b).

On examination the patient was macrocephalic with OFC of 59 cm (>97th centile). His facial features appeared similar to his mother with broad forehead, deep set eyes, broad nasal root, prominent columella, and short philtrum (Fig. 2a). Poor eye contact, low attention span and motor stereotypies were observed. There were no neurocutaneous markers and his neurological examination was grossly normal. Standardized psychological assessment could not be completed due to limited attention for directed tasks and his gross cognitive testing indicated his functioning at 25-month-old level. His past evaluation included normal MRI of Brain, EEG and negative fragile X testing.

Patient 2 is a now 3-year-old boy who presented for genetic evaluation due to global developmental delay and congenital anomalies. His parents were non-consanguineous and of mixed ethnicity (Fig. 2c). Both parents are generally healthy and had unremarkable developmental histories. His birth weight 2.81 kg (13th percentile), length 49 cm (32nd percentile) and OFC of 32.1 cm (3rd percentile). Cutis aplasia was found after delivery. He was noted to have a large, irregular skin defect on his posterior scalp with some islands of normal skin. The calvarium in the affected region was thin. Three stellate shaped ulcerations involving vertex and occiput of the scalp were noted (largest measuring approximately 4 cm × 3 cm; the next smallest measuring approximately 3.5 cm × 2.5 cm; and the smallest approximately 1 cm in diameter). The scalp defect was managed conservatively and healed with scarring and patchy alopecia. At 3 years of age he had predominant language delay and lagging behind in his head circumference for age. Examination revealed OFC of 46 cm (<3rd percentile). Scalp was notable for extensive scarring and patchy alopecia. Ears were cupped and mildly simplified. He had broad forehead, hypertelorism with arched eyebrows and a slightly upturned nasal tip. Neurological examination was unremarkable except for expressive language delay. Previous genetic testing included a normal chromosome microarray analysis.

### Genetic analysis

Patient 1 had exome sequencing performed through a CLIA-certified laboratory. A c.77C > A, p.(Ser26*) pathogenic nonsense variant was reported in *TRIO* (NM_007118.2). This variant was inherited from the patient’s affected mother. This variant has not been previously reported in gnomAD, nor has it been previously associated with a disease phenotype based on ClinVar or published literature. It is located in exon 1 and would cause truncation in the n-terminal SEC14 domain which lies upstream of the spectrin repeats. In silico prediction of NMD suggests this variant would cause the affected transcript to be targeted for degradation [40]. The function of the SEC14 domain has not been well established other than a role in subcellular targeting and possibly the regulation of endosome dynamics [41]. Patient 1 also had a c.11G > C, p.(Arg4Pro) missense variant of uncertain significance (VUS) reported in *PIK3CA* (NM_006218.2). This variant was also maternally inherited. Due to lack of phenotypic overlap with the associated MCAP syndrome, CLOVE syndrome, and fibroadipose hypoplasia, this variant was not thought to be contributing to the patient’s phenotype.

Patient 2 had exome sequencing performed through the same clinical laboratory as patient 1. A c.2690dupA, p.(Gln898Alafs*61) pathogenic frameshift variant was reported in *TRIO* (NM_007118.2). The variant was not inherited from the unaffected mother and a paternal sample was not available. This variant has not been previously reported in gnomAD, nor has it been previously reported in ClinVar or published literature. It is located in exon 15 and would cause an early truncation in the spectrin repeats. In silico prediction of NMD also suggest this variant would cause any affected transcript to be targeted for degradation [40].

## Discussion and conclusions

Here we report on two patients with novel pathogenic presumed LOF variants reported in *TRIO*. Both patients have phenotypes overlapping with previously reported patients including severe (patient 1) and moderate (patient 2) developmental delay, and ID. Both had dysmorphisms including broad/high forehead, deep set eyes, broad nasal root, prominent columella and short philtrum in patient 1 and cupped ears, broad forehead, hypertelorism, arched eyebrows and a slightly upturned nasal tip in patient 2. Of note, Ba et al. also reported several patients with high forehead in their cohort [22]. A novel phenotype presenting in patient 2 includes extensive cutis aplasia which has not been previously reported and may represent an expanded phenotype associated with this disorder.

Microcephaly was reported in patient 2 which matches well with most previously characterized patients with nonsense/truncating variants (including p.(Arg217*), p.(Gln768*), p.(Asp1251Valfs*11), p.(Trp1376*), p.(Gln1489Argfs*11), p.(Arg1620Serfs*10), p.(Val1698Leufs*61), p.(Lys1918*), p.(Leu2031Phefs*9), and p.(Phe2472Serfs*54)) [16, 22, 35–37]. Notably, patient 2 does not show a severe ID phenotype or macrocephaly as is reported for patients with missense variants in the same spectrin repeat domain, reinforcing that microcephaly is associated with the majority of presumed LOF variants and thereby with haploinsufficiency of the gene.

Interestingly, patient 1 had macrocephaly in contrast to the majority of other patients with truncating and presumed LOF variants. Barbosa et al. described a group of non-related individuals with macrocephaly who had missense variants clustering on the spectrin repeat domain. The authors performed in vitro experiments and showed that the missense variants at the spectrin repeat 7 cause a gain-of-function effect, inducing hyperactivation of RAC1. None of the individuals with presumable LOF variants in their cohort presented with macrocephaly [36]. One possibility as to why patient 1 seems to have a different phenotype may be that the variant is in the far N-terminal region and therefore there are several transcripts with downstream alternate start sites that could be unaffected by this variant (ENST00000509967.2, ENST00000513206.1, ENST00000344135.5). Variations in the location and timing of expression of these alternate transcripts may explain the discrepancy in observed phenotypes between patient 1 and other patients with presumed LOF variants.

Overall, we demonstrate two cases with patients presenting with neurological phenotypes and reported pathogenic, presumed LOF variants in *TRIO*. We add to the known clinical phenotype spectrum associated with this gene with the report of cutis aplasia in patient 2. We also add to the literature the third documented case of familial autosomal dominant inheritance of a *TRIO*-associated disorder highlighting the variable expressivity associated with *TRIO* disorders. Moreover, we report on a patient with macrocephaly and a nonsense variant upstream to the domain hotspot that has been associated with this phenotype. Future work clarifying the status of transcript degradation/protein translation for truncating and nonsense variants in patient samples, especially testing for hyperactivation of RAC1 in the individual with a nonsense variant is needed to fully characterize the effect of these types of variants.

## Data Availability

Genomic sequence data cannot be deposited due to restrictions imposed by the IRB. The data that support the findings of this study are available on request from the corresponding author. The data are not publicly available due to their containing information that could compromise the privacy of research participants.

## Abbreviations

TRIO: Triple functional domain
GEF: Guanine nucleotide exchange factor
ID: Intellectual disability
ASD: Autism spectrum disorder
OFC: Occipitofrontal circumference
NMD: Nonsense-mediated decay
LOF: Loss-of-function
AD: Autosomal dominant
HGVS: Human Genome Variation Society
